# On the Reputed Causes of Yellow Fever, Etc.

**Published:** 1854-08

**Authors:** 


*On the Reputed Causes of Yellow Fever* and the so called Sanitary Measures of the Day, by M. MORTON DOWLER, M.D., Fourth District, New Orleans.

THIS is the title of a pamphlet of 20 pages reprinted from the New Orleans Medical Journal. Dr. Dowler's object is to show:

First, that the first cases of yellow fever in 1853 occurred in a portion of the city entirely cut off from the shipping, and several days before any cases had been discovered on board of any of the ships in port.

Secondly, that the first cases had not been contracted by contact with persons laboring under yellow fever.

Thirdly, that the disease was not communicated by these first cases to any other person.

Our author battles against the theory that filth is the producing cause. He shows that those parts of the city containing the largest quantities of decaying vegetable and animal matter did not suffer more than those where the most scrupulous regard was paid to cleanliness. In addition to the facts adduced the following argument is given:

"In fact, the filth theory is wholly untenable, not only from the entire history of yellow fever in this community, but from every analogy known to science. Every product, whether solid, fluid, or aeriform, or imponderable, whether resulting in the decomposition of animal, vegetable, or mineral matter, produces effects which are remarkably uniform. They do not discriminate between the acclimated and the unacclimated, nor are their noxious or innocuous properties at all modified by such circumstances. For instance the deposits at Gormly's Basin give off amongst other substances, sulphuretted, carburetted, and phosphoretted hydrogen, ammoniacal gases, substances which with ordinary ventilation merely give evidence of their existence by offending the nostrils, in bad ventilation become oppressive, and when highly concentrated quickly destroy life. The effects are uniform in all climates, and to assume that the mere enhaling of a mixed atmosphere, which one person breathes with perfect impunity, should give another the yellow fever, another the cholera, and a third the typhus fever, it is simply a *reductio ad absurdum*. Our knowledge of these gases demonstrates that the reputed cause is insufficient to produce the effect, and there is not a shadow of proof to show that it does produce it. The exhalation from departed animal and vegetable life mingle with our every breath, and it has never been shown, nor ever can be shown that they act otherwise than uniformly. Touching the filth question, let us not be misunderstood. Let every alderman "search the scriptures." There is scarcely a chapter in that sacred book which does not admonish all to "wash and be clean." Common decency also speaks aloud. False and improbable theories, however, can do nothing else than lead to useless and extravagant and corrupt legislation, and to lull the public into a false security.

